# Beyond Marine Reserves: Exploring the Approach of Selecting Areas where Fishing Is Permitted, Rather than Prohibited

**DOI:** 10.1371/journal.pone.0006258

**Published:** 2009-07-22

**Authors:** Natalie C. Ban, Amanda C. J. Vincent

**Affiliations:** 1 Project Seahorse, Fisheries Centre, The University of British Columbia, Vancouver, British Columbia, Canada; 2 Australian Research Council Centre of Excellence for Coral Reef Studies, James Cook University, Townsville, Queensland, Australia; Monash University, Australia

## Abstract

**Background:**

Marine populations have been declining at a worrying rate, due in large part to fishing pressures. The challenge is to secure a future for marine life while minimizing impacts on fishers and fishing communities.

**Methods and Principal Findings:**

Rather than selecting areas where fishing is banned – as is usually the case with spatial management – we assess the concept of designating areas where fishing is permitted. We use spatial catch statistics for thirteen commercial fisheries on Canada's west coast to determine the minimum area that would be needed to maintain a pre-ascribed target percentage of current catches. We found that small reductions in fisheries yields, if strategically allocated, could result in large unfished areas that are representative of biophysical regions and habitat types, and have the potential to achieve remarkable conservation gains.

**Conclusions:**

Our approach of selecting fishing areas instead of reserves could help redirect debate about the relative values that society places on conservation and extraction, in a framework that could gain much by losing little. Our ideas are intended to promote discussions about the current status quo in fisheries management, rather than providing a definitive solution.

## Introduction

The oceans have suffered declines in faunal biomass and biodiversity [1], [2], [3], with fisheries constituting the single biggest human-induced pressure on marine life [4]. Marine reserves (no-fishing zones) have been widely hailed as providing one powerful tool – but not a panacea – for halting the decline of overexploited fish and invertebrate populations [5], [6], [7], [8], [9]. The evidence that they increase biomass, abundance, and average size of exploited organisms within their boundaries [5], [6] has prompted international commitments to establish marine protected areas (including reserves) under the Convention on Biological Diversity and at the World Summit on Sustainable Development [10], [11]. Nevertheless, and despite this accord on the value of marine reserves, they are being implemented far too slowly to meet agreed targets for marine protection [10]. Their impacts on fisheries remain uncertain, and the extent of fisheries benefits, if any, varies [9], [12].

Given the slow accumulation of marine reserves relative to international targets, we turn the problem on its head [e.g., 13]. We embrace the challenge of presuming that the entire ocean is initially protected from fishing rather than open to fishing [14], [15], [16]. At present, fisheries exploitation is specifically excluded (i.e., areas are protected) in less than 1% of the oceans [10]. Given biodiversity concerns and the challenging task of managing fisheries with limited data, it is increasingly vital to explore ways to restrict fisheries spatially while respecting their socioeconomic and nutritional contributions. Such restrictions should, ideally, also meet systematic conservation planning criteria of representation and persistence [17].

Conceptually the approach of selecting fishing areas is very similar to using fisheries as a “cost” to represent economic losses to fisheries in marine reserve selection [18], [19]. “Cost” in this context refers to the socio-economic or political cost of adding an area to a marine reserve [20]. The typical approach in marine reserve selection is to ensure representation of biodiversity features whilst minimize the cost to fisheries. By treating fisheries as a cost, marine reserve selection tools require that they be summarized in one layer – this often involves adding or averaging the catches, effort, or catch-per-unit-effort of fisheries for each area [20]. By targeting fisheries instead of treating them as a cost, we select the most productive fishing regions while minimizing the area fished. The main advantage of this approach is that each fishery can be selected for individually, thereby ensuring that all fisheries would be able to continue.

The goal of our research was to explore the possible conservation gains that might accrue from different hypothetical levels of restriction on fisheries. We use data from the Pacific coast of British Columbia, Canada (approximately 49°N to 54°N latitude) for this initial foray. Our purpose is not to provide a definitive answer to fisheries management or reserve selection. Rather, we seek to promote discussion about the current status quo of our ocean management approaches.

## Results

**Figure 1:**
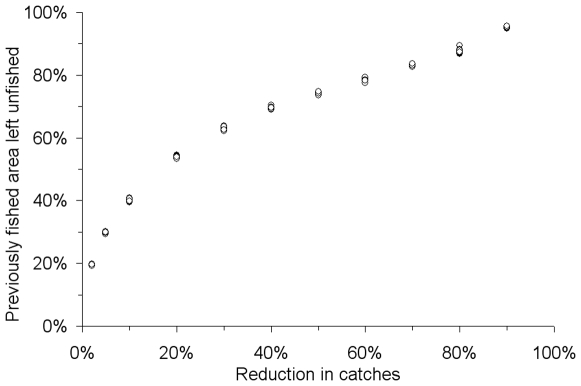
Decreases in areas fished resulting from reductions of catches for 13 commercial marine fisheries (British Columbia, Canada). Each of 11 scenarios was repeated 10 times, with 100 runs of one million iterations each (11,000 runs). The result requiring the least area of each of the 10 repetitions per scenarios is graphed (i.e., there are 10 data points per scenario; some overlap closely and appear as one).

**Figure 2:**
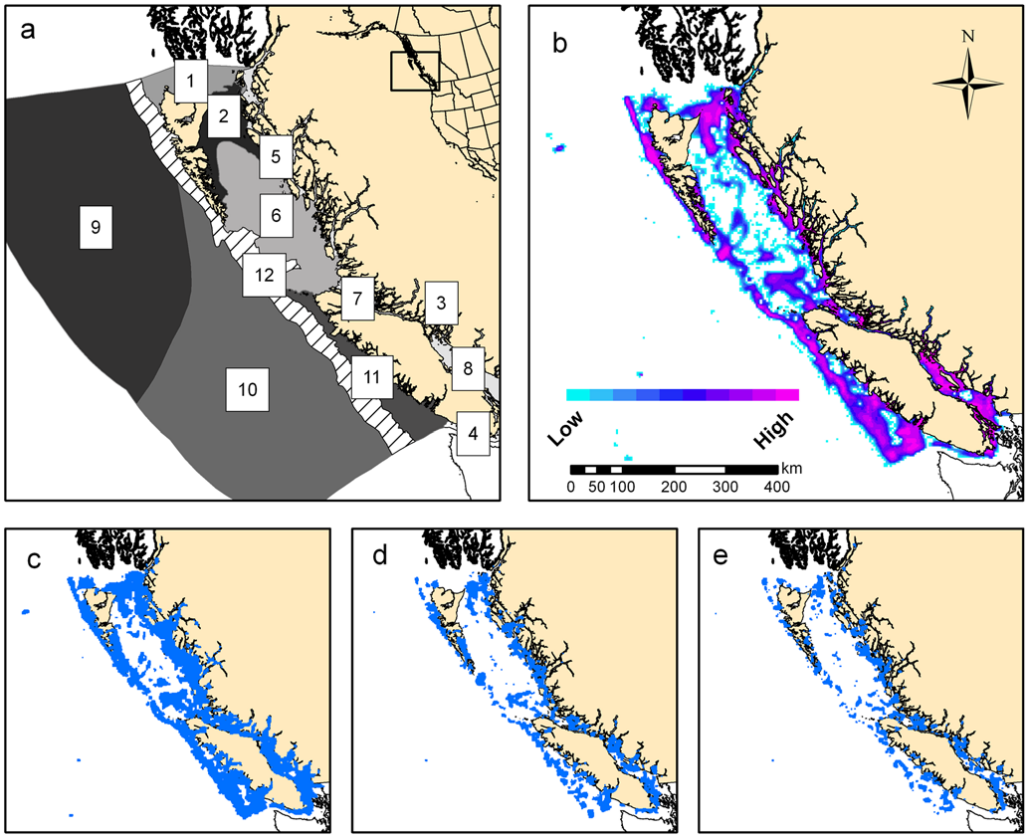
Marine ecosections in British Columbia and selected permitted fishing area solutions. The marine ecosections (a) are 1 = Dixon Entrance; 2 = Hecate Strait; 3 = Johnstone Strait; 4 = Juan de Fuca Strait; 5 = North Coast Fjords; 6 = Queen Charlotte Sound; 7 = Queen Charlotte Strait; 8 = Strait of Georgia; 9 = Subarctic Pacific; 10 = Transitional Pacific; 11 = Vancouver Island Shelf; 12 = Continental Slope. The selection frequency map (b) shows the importance of areas to commercial fisheries based pm the summed solution results from Marxan. The permitted fishing area solutions (in blue) are for a sample of the scenarios that minimize the area fished with the corresponding percent reduction in commercial fishing catches: (c) 5%, (d) 20%, (e) 40%.

Our analyses show that very small reductions in fisheries yields – if allocated in a strategic manner across space – can offer promising conservation benefits in both space and composition. For example, catch reductions of only 2%–5% could result in no-fishing areas constituting 20% or 30% of previously fished areas (Fig. 1). Every subsequent reduction in target catches yielded yet larger no-fishing areas (Fig. 1 and Fig. 2). Moreover, for each scenario, the multiple solutions that released the greatest area from fishing (Fig. 2) described no-fishing areas that included representation from all twelve ecosections in British Columbia (Table 1); these ecosections delineate marine regions based on physical criteria. Maintaining catches at 95% of recent levels (or more, depending on the fishery) resulted in no-fishing areas that protected at least 17%, and an average of 55%, of each physical and habitat feature (Table 2). In this scenario the total area protected would be 30% in exchange for a mean 4.6% reduction in catches (Table 3).

**Table 1:** Gap analysis by ecosection for the most spatially limited result for each scenario.

	Area of ecosection (ha * 1000)	% of area fished	Percent reduction in catches (italics), resulting in percent protected (plain, in %)										
			*2%*	*5%*	*10%*	*20%*	*30%*	*40%*	*50%*	*60%*	*70%*	*80%*	*90%*
Continental Slope	3,330	53.8	55.3	60.0	64.8	74.5	78.6	79.2	84.5	88.5	91.9	89.6	97.2
Dixon Entrance	1,089	55.4	57.8	64.8	72.0	79.1	83.0	90.8	86.9	89.9	95.3	93.6	98.1
Hecate Strait	1,280	77.0	36.5	43.0	50.0	57.6	64.9	70.9	74.5	82.7	91.7	85.5	95.5
Johnstone Strait	239	98.0	11.4	19.2	24.1	46.3	58.1	65.1	98.0	73.0	81.7	81.8	96.1
Juan de Fuca Strait	150	90.8	15.5	27.0	67.4	56.7	74.6	72.8	69.5	96.6	100	97.7	100
North Coast Fjords	958	91.9	23.6	33.9	46.9	59.6	68.7	72.8	80.5	83.3	91.3	85.2	96.4
Queen Charlotte Sound	3,642	55.7	60.7	68.1	75.9	82.5	87.5	89.1	89.8	90.1	97.4	94.2	99.2
Queen Charlotte Strait	220	94.5	7.9	18.1	33.2	46.2	45.6	69.4	66.4	56.8	93.2	74.6	86.0
Strait of Georgia	815	94.8	7.9	63.0	14.6	27.3	31.2	51.7	50.7	64.5	79.4	77.8	92.7
Subarctic Pacific	17,098	0.3	99.8	99.9	99.9	99.9	99.9	100	99.6	100	100	100	100
Transitional Pacific	14,850	0.1	100	100	100	100	100	100	100	100	100	100	100
Vancouver Island Shelf	1,670	89.2	17.8	24.4	30.9	42.2	56.3	66.8	70.7	76.4	87.9	75.5	93.8

**Table 2:** Detailed analysis of the result of the 5% catch reduction scenario that produced the greatest area unfished, indicating ecosystem components that would be protected.

		Total area (ha) of each ecological feature	% outside permitted fishing areas
Depth	Shallow (0–20 m)	743,853	40.6
	Photic (20–50 m)	1,521,555	42.9
	Mid-depth (50–200 m)	60,400,258	94.3
	Deep (200–1000 m)	3,469,678	43.9
	Abyssal (>1000 m)	33,627,695	99.7
Temperature (summer at seabed bottom)	Warm (9–15°C)	2,438,557	32.9
	Cool (<9°C)	42,820,022	88.0
Slope	Flat (0–5%)	40,556,889	87.2
	Sloping (5–20%)	4,737,411	67.4
	Steep (>20%)	42,749	43.0
Current	High (>3 knots)	212,713	39.0
	Low (<3 knots)	45,162,974	85.3
Substrate	Mud	2,295,529	27.6
	Sand	4,852,577	47.7
	Hard	3,631,788	53.5
Exposure	High	42,616,399	89.0
	Moderate	1,287,192	17.4
	Low	1,470,964	30.4
Relief	High	206,158	17.6
	Moderate	20,839,047	93.9
	Low	43,040,993	87.7
Salinity (annual average at surface)	Mesohaline (5–18ppt)	147,957	22.1
	Polyhaline (18–28 ppt)	11,279,517	91.7
	Euhaline (28–33 ppt)	43,945,636	87.0
Stratification	Mixed	4,931,996	36.8
	Weakly-mixed	2,083,666	42.3
	Stratified	37,823,783	94.4
Kelp		79,806	19.5
Eelgrass		10,449	28.7
Clam		18,978	22.5
Herring spawn		99,737	22.3
Sponge reefs		69,733	85.0

**Table 3:** Actual catch reductions and estimated direct financial impact for each fishery under the scenario that (a) reduced overall catch by 2%, 5% and 10% and (b) produced the greatest area unfished at that level.

	Actual catch reduction (%)			Estimated direct impact (US$)*		
Commercial fishery	2% catch reduction scenario	5% catch reduction scenario	10% catch reduction scenario	2% catch reduction scenario	5% catch reduction scenario	10% catch reduction scenario
Crab	2.0	5.0	10.0	665,624	1,664,103	3,327,551
Geoduck	2.0	5.0	10.0	113,128	282,906	565,828
Green urchin	2.0	5.0	10.0	5,021	12,512	25,122
Groundfish trawl	2.0	5.0	10.0	470,332	1,175,818	2,351,668
Krill	1.9	4.7	9.9	NA	NA	NA
Prawn	2.0	5.0	10.0	234,238	585,538	1,171,154
Red urchin	2.0	5.0	10.0	186,231	465,469	932,242
Sablefish longline	1.2	1.5	3.4	35,061	42,496	95,713
Sablefish trap	2.0	5.0	10.0	266,145	665,345	1,330,782
Schedule two	2.0	5.0	10.0	19,455	48,632	97,280
Sea cucumber	2.0	4.7	10.0	35,661	83,147	178,338
Shrimp trawl	2.0	4.2	7.2	207,272	430,335	748,438
ZN catch	2.0	5.0	10.0	17,223	43,057	86,118
Total				2,255,391	5,499,359	10,910,233

* Ex-vessel data obtained from the Sea Around Us online database (www.seaaroundus.org) [21]. We used mean annual prices from 2000 to 2004 in the reporting units of US$. At the time of writing the Canadian and US currencies were about par.

NA = ex-vessel data not available.

Estimates of the direct losses for the 2%, 5%, and 10% reduction scenarios based on ex-vessel prices [21] revealed that the combined cost to fisheries ranges from US$2.3 million per year to US$11 million per year for the above scenarios (Table 3). While this is less than one percent of British Columbia's seafood industry – which is valued at $1.4 billion annually – and an even smaller portion of British Columbia's oceans economy – valued at $11.4 billion annually [22] – it could be significant for some fisheries. In addition, the approach we cite would result in spin-off losses (*e.g.*, job losses in the seafood processing industry).

The approach we employed for selecting permitted fishing areas used catches averaged over multiple years as the input, yet the result of the 5% reduction scenario also performed well when analyzed using documented annual catches for geoduck, green urchin, red urchin and sea cucumber fisheries (Table 4). As expected, we found some inherent spatial and temporal variability in the proportion of catches that would fall within the permitted fishing areas each year. The greatest range for a target of 95% of catches retained across all fisheries was a 2–12% reduction in sea cucumber catches, depending on the year.

**Table 4:** Proportion of annual commercial fisheries catches that fall within the permitted fishing area result of the 95% target scenario.

Fishery	Annual data	Average	Standard deviation	Minimum	Maximum
Geoduck	2002–2004	95.04%	1.91%	92.96%	96.73%
Green urchin	1998–2003	94.29%	2.89%	90.49%	97.05%
Red urchin	1997–2003	95.07%	0.39%	94.72%	95.82%
Sea cucumber	1997–2004	94.81%	3.07%	88.25%	97.97%

## Discussion

### Potential conservation and fisheries benefits of permitted fishing areas

The practical approach used in this study allows for explicit analyses of trade-offs between small reductions in fisheries – in a spatially strategic manner – and large gains for marine conservation through spatial protection. Managing marine environments by selecting permitted fishing areas rather than marine reserves would represent a much-needed paradigm shift in areas where little headway is being made in marine reserve establishment. Instead of debating the merit of each potential marine reserve, the discourse could focus on analyses of the ecological benefits of small reductions in fishing.

This approach has the potential to offer real conservation benefits. At a minimum, the approach outlined here would protect the same proportion of fished populations as the target reduction in catches, assuming even catchability in space. Because we suggest reducing quotas by the target percentage used for the spatial selection of permitted fishing areas, effort within these areas would not increase. Therefore any alterations to source-sink dynamics would already have occurred with previous fishing patterns. Even small marine reserves that protect only a fraction of populations have been shown to increase the size, number, and diversity of fish within their boundaries [5], [23], [24]. Given the usually exponential increase in fecundity of fishes that grow larger within protected areas, protecting even a small proportion of the population could greatly enhance numbers in areas that continue to be fished. By changing the reserve to fished ratio, recruitment effects could be significantly greater than anything seen to date. For larger species, fisheries yields may respond to no-fishing areas with a response that exceeds the results of conventional fisheries management by 60 % [25]. However, fecundity has not increased in all closed areas [26], and hence the effects of marine reserves can be unpredictable.

Even though ecological goals were not included *a priori* in the designation of the permitted fishing areas, the areas that fell outside permitted fishing areas included good representativeness across ecosections [27] (Table 1). Further detailed analysis of the scenario with 5% catch reduction showed that the areas outside the permitted fishing area represented key physical and habitat features (Table 2).

Even while protecting large (and representative) tracts of ocean, the proposed approach of designating permitted fishing areas could reasonably be expected also to strengthen fisheries in three ways if the experience in some marine reserves holds [e.g.], [6], [23,28]. First, the removal of destructive fishing gear from the areas outside the permitted fishing areas should promote improved habitat quality [29], while also reducing bycatch [30]. Second, given the benefits of even small reserves for population recovery [28], the areas outside the permitted fishing areas could be enhanced by fish populations within permitted fishing areas [6], [31]. Third, many fisheries around the world are operating unsustainably [32], such that reductions in catch while setting permitted fishing areas could also move these fisheries closer to desired biological reference points for sound management [33]. Such changes might well offset catch reductions over the long run.

We are well aware that the actual effects of permitted fishing areas are untested and hence uncertain. Clearly and importantly, our estimates of direct losses to fisheries need refinement to achieve greater realism. Our approach is simplistic in that we currently use ex-vessel prices and production, and hence assume that productivity is proportional to profitability. Models of fishing behaviour, and analyses of current allocation rights and dependencies, should be developed to provide better estimates of the potential costs to fisheries, such that they can be incorporated into the analysis of conservation benefits. A more advanced version of our analyses would also incorporate other commercial fisheries, recreational fisheries, timing of fishing effort, and more detail on ecologically important areas. Ironically, launching the assessment process we propose – in a consultative fashion – might be a particularly effective way of eliciting or prompting the collection of just such important data, which are seldom available (or at least publicly accessible) in even the best resourced management jurisdictions.

The flexibility of the approach used here could help to enhance societal acceptance of and compliance with spatial planning, particularly among fishers. The decision support tool we used – Marxan – facilitates decision-making, without making decisions. Indeed, because it offers multiple solutions that may differ only slightly in their efficiency, the exact choice of permitted fishing areas can be adjusted for social acceptability and ecological viability [34]. Fishers' input will be important in setting commercial catch targets by fishery, verifying formal data [35], mapping and scaling fisheries that lack formal spatial data, and in agreeing to the permitted fishing areas. By using Marxan to set catches as targets instead of as a cost – the common approach in reserve selection – each fishery is targeted the same way, and therefore (under the assumptions of this approach) would incur a loss proportional to that fishery. In contrast, when all fisheries are combined into one cost, some fisheries may be disproportionately affected.

The approach of selecting permitted fishing areas would be expected to yield useful results in other geographic areas. Gear types used in British Columbia are typical of commercial fisheries elsewhere – trawl, hook and line, gillnet, seine, trap, and dive – and bioeconomic models suggest consistency in fisher behavior across locations [36]. Moreover, modeling has previously shown that optimal harvesting strategies always include marine reserves for populations with sedentary adults, even before consequent improvements in habitat recovery are considered [37]. Trials of this approach must, however, be taken elsewhere to determine whether, for example, the resultant no-fishing zones are generally ecologically representative.

### Assessing the costs and benefits

While optimistic about the potential of our approach, we are well aware that many challenges remain to be resolved. First, we would benefit from knowing more about the connectedness of the fished and unfished area over space and time, in order to understand responses to spatial management; this is also true of MPA design and conventional management tools. Second, some fisheries in the portfolio that already operate sustainably might gain few benefits from the spatial management we propose, and would essentially be making concessions for other fisheries and/or for broader conservation principles. Third, our approach focuses only on fisheries (and only commercial fisheries in this trial study), whereas other marine and terrestrial uses also significantly affect the ocean. Fishing, however, is the main threat, and hence a tangible starting place for making conservation gains. Fourth, the large no-fishing zones arising from our approach, might lead to claims that no further areas need be protected, whatever their claims for conservation. Fifth, it remains to be determined whether the areas protected through this approach would provide the same conservation benefits conventional marine reserve selection. We do, however, know that both approaches tend to lead to protection for areas that are less valuable economically.

As ever, no single management measure will achieve all goals. The effectiveness of our approach in terms in accelerating protection will depend, in large measure, on the extent to which fishers gain yields in proportion to the benefits they cede in the no-fishing zones. Some conflict is still likely if, for example, the best fishing grounds – and hence the areas most likely to be included in permitted fishing areas – are also (a) the most sensitive habitats with the highest fish densities or (b) the most sensitive habitats even if they do not have high fish densities. These areas would ideally be protected in no-fishing zones, to the annoyance of fishers. Worse, however, would be to leave them in the permitted fishing areas, where they might come under more concentrated fishing unless quotas were reduced commensurate with the spatial contraction of the fishery. In terms of spatial management, the best approach is likely to combine the selection of permitted fishing areas with the identification and protection of sensitive habitats, whilst respecting the needs of fishers with limited ranges, such as small-scale fishing fleets or anglers. In addition, conventional fisheries management approaches will continue to be essential to managing fisheries within fished areas, especially to reduce the race to fish, realign incentives to reduce bycatch and habitat damage, and promote profitable fisheries; these are some of the roots of fisheries management failures [9].

The available of fishing data will influence the completeness of the selection of permitted fishing areas. In particular, historical fishing data are rarely available. In some regions, the areas receiving less fishing effort – areas more likely to fall outside of permitted fishing areas – might be depleted through past fishing. Some marine reserves sited in degraded and depleted areas have shown remarkable recovery [38], and therefore such areas likely have the capacity to recover. In the case of British Columbia, areas that have been fished the most historically – those close to population centers – are still among the most heavily fished, and hence sequential exhaustion of past fishing sites is less likely to be an issue than in other regions. In regions where spatial fishing data are not available – such as most small-scale fisheries – interviews with fishers could be used to collect such data.

Approaches such as ours are unlikely to succeed without strong community and political support. Given that selecting permitted fishing areas would restrict fishers' flexibility in where to fish, support for the concept is not guaranteed. However, many fisheries are continuing to decline, and some scientists are calling for large reductions in catches [39]. By specifying targets for each fishery based on sustainability estimates and permitting all fisheries in the same areas, ecological benefits may be greater than by managing each fishery independently.

The designation of permitted fishing areas will face many of the same obstacles as in the selection of marine reserves. First, there are data availability issues and knowledge gaps. In both cases, we usually lack spatial data for at least some fisheries, biological and range data for at least some species, and an appropriate understanding of dispersal and connectivity [40]. Given this lack of spatially structured data, specific modeling of the anticipated increases and decreases in species and ecosystem dynamics may be a challenge. Second, similar implementation and management issues might arise for permitted fishing areas and marine reserves. Enforcement would still be a challenge, and the political will to proceed with establishment has to exist for advances to be made.

### Conclusion

Given the dismal state of many fisheries, time is ripe to debate alternative approaches. We have little to lose – and much to gain – in trying a new approach in areas where marine conservation advances have been inadequate. It appears, *ab initio*, that large areas that are representative of ecoregions and habitats might be protected at a small cost to fisheries (although particularly sensitive areas may have to be included *a priori*). Moreover, the dependency of the approach on explicit commercial catch targets for each fishery forces us to define the trade-offs we are willing to make to ensure a healthy ocean. The alternative to the approach described here seems to be the continuation of the *status quo*, which has resulted in the sequential collapse of fisheries [1], [41] with only a small proportion of the ocean protected by marine reserves. At a minimum, a debate about management assumptions is much needed, if innovative approaches are to emerge.

## Methods

### Selection of Permitted Fishing Areas: The decision support program used

We applied Marxan [42], [43], a decision support tool that has commonly been used to plan reserves, to spatial catch statistics for 13 commercial marine fisheries in British Columbia, Canada. Marxan tries to find the least expensive solution to the following objective function, using a simulated annealing algorithm [44]:

Total score  =  ∑ planning unit cost + (boundary length modifier * ∑ boundary cost) + feature penalty

We created a grid of 2 km by 2 km cells (with each cell considered to be one planning unit), to cover the study area, populated it with the spatial catch data, and then ran scenarios to select fishing areas. As spatial catch data were not available for recreational fisheries, our analysis is limited to commercial fisheries. We set the boundary length modifier – which controls the boundary to area ratio of the Marxan output – high enough so that the results were spatially compact. We pre-specified targets for commercial catches (kg) for each fishery, then set the penalty factor high enough to ensure that were met. Marxan provides a good approximation to an optimal solution by incorporating a random component to adding and removing planning units. Rather than settling on a single outcome, Marxan produces many solutions for any target that is proposed. The frequency with which particular planning units are chosen across different solutions is a measure of how important those planning units are for meeting the commercial catch targets efficiently.

### The data

We obtained spatial catch data from Fisheries and Ocean Canada for 13 commercial fisheries in British Columbia, Canada. The scale of analysis – the province of British Columbia – matches the scale of management. For confidentiality reasons, eight sets of data had been summarized in 4 km by 4 km grids: ZN fishery (hook and line inshore rockfish), shrimp trawl, schedule 2 (hook and line, other species), sablefish trap, sablefish longline, prawn trap, groundfish trawl, and crab. Similarly, the other five sets of data had been grouped into 10 km by 10 km grids: sea cucumber, red urchin, krill, green urchin, and geoduck. Many of the commercial fisheries have a high percentage of observer coverage and have vessel monitoring systems; we therefore believe that the data are as reliable as catch data can be. If fishing data are to be used for the purpose of selecting fishing areas, workshops with managers and fishers could be used to assess the reliability of the data. Also, if the fishing industry knows that the data could be used for such a purpose, reliability of the data may improve.

We normalized all data to the average annual catch (kg) for each planning unit. Data were averaged per annum over the temporal duration of spatial data collection, which extended between 3 and 12 years for any given fishery (1993–2004). We used 2 km by 2 km planning units that assumed even spatial distribution of catches for each original 4 km by 4 km or 10 km by 10 km grid.

### The scenarios and analyses

We set each of 11 scenarios to maintain a particular target level of recent mean annual commercial catches (kg), from 98% to 10%; the targeted yield reductions thus ranged from 2% to 90%. We repeated each scenario ten times, with 100 runs of one million iterations each. The results for each scenario integrated all 13 fisheries, with each fishery maintaining at least that target catch.

We carried out a detailed assessment of the run with a target of a 5% reduction in catches (in all fisheries) by examining the proportion of different habitat types or surrogates that fell within the areas where fishing was allowed to continue (permitted fishing areas). Our intention was to determine which features would be protected by selecting permitted fishing areas, and which would remain unprotected. The habitat types were described by depth, exposure, relief, slope, current, temperature, substrate, salinity and stratification. In addition, limited spatial information was available for the distribution of kelp, eelgrass, herring spawn areas, and clam beds.

We further assessed the performance of the run with targeted 5% reduction in catches on annual spatial catch data for the four commercial fisheries for which we had annual data: geoduck, green urchin, red urchin and sea cucumber. Furthermore, we took the scenarios for the 2%, 5% and 10% reduction that resulted in the least area fished, and assessed the predicted reduction in catches, and the expected losses based on ex-vessel prices, for all 13 fisheries.
